# *Candida albicans* Biofilm Inhibition by Ethnobotanicals and Ethnobotanically-Synthesized Gold Nanoparticles

**DOI:** 10.3389/fmicb.2021.665113

**Published:** 2021-05-24

**Authors:** Khristina G. Judan Cruz, Eleonor D. Alfonso, Somar Israel D. Fernando, Kozo Watanabe

**Affiliations:** ^1^Department of Biological Sciences, College of Science, Central Luzon State University, Science City of Muñoz, Nueva Ecija, Philippines; ^2^College of Agriculture, Nueva Ecija University of Science and Technology, Nueva Ecija, Philippines; ^3^Department of Civil and Environmental Engineering, Ehime University, Matsuyama, Japan; ^4^Center for Marine Environmental Studies (CMES), Ehime University, Matsuyama, Japan

**Keywords:** *Candida*, biofilm, quorum sensing, gold nanoparticles, ethnobotanicals, Bcr1, HSP90

## Abstract

The virulence and drug resistance of globally prevalent *Candida albicans* has presented complications toward its control while advances in effective antivirulence drugs remain critical. Emerging methods are now being evaluated to facilitate development of novel therapeutic approaches against this pathogen. This study focuses on the biofilm formation inhibition of ethnobotanical crude extracts and the use of nanotechnology through the ethnobotanically-synthesized gold nanoparticles to control *C. albicans*. Control on biofilm formation was compared using crude extracts (CEs) and biologically synthesized gold nanoparticles (CEs + AuNPs). Significantly lower biofilm formation was exhibited in thirteen (13) CEs and fourteen (14) CEs + AuNPs. Biofilm-linked genes *Bcr1* and *HSP90* expression were consequently downregulated. Higher biofilm inhibition activity was noted in some CEs + AuNPs compared to its counterpart CEs. This study emphasizes the biofilm inhibition activity of ethnobotanicals and the use of nanoparticles to enhance delivery of compounds, and points to its prospects for developing anti-pathogenic drugs without evolving resistance.

## Introduction

Candida albicans is a globally prevalent pathogen owing to its capability to survive at diverse biotic and abiotic sites (Mathe and Van Djick, 2013; Gao et al., 2016; Lee et al., 2018) with the ability to cause an array of infections that ranges from superficial to life threatening. Candida infections, commonly known as candidiasis or candidosis (Douglas, 2003; Moran et al., 2012), can occur as a consequence of its ability to develop a biofilm, a quorum sensing (QS) mechanism associated with its pathogenicity (Silva et al., 2011; Pfaller, 2012). The complexity of its biofilm offers virulence through its three-dimensional structure and innate drug resistance (Pereira et al., 2020) while withstanding host immune responses (Lee et al., 2018), thus, requiring multifaceted scheme for its control.

This high level of tolerance to multiple drugs (Duo et al., 2010; Gao et al., 2020) contribute complications to control this pathogen, and this pose serious threats to healthy and medically compromised individuals that often lead to severe fatal infections (Hall-Stoodley et al., 2004; Lohse et al., 2018). Control of *Candida* through commercial antifungal drugs such as triazole drugs and echinocandins (Cruz et al., 2007; Peman et al., 2009) is practiced, but not without the consequences of developing fungal resistance and reduced susceptibility. Administration with these antifungal drugs inevitably produce evolving strains throughout long-term treatment (Cruz et al., 2007), hence, the increasing incidence of drug-resistant *Candida*.

To address emerging virulence and fungal resistance, Quorum Sensing (QS) mechanisms are now targeted. Quorum sensing is a cell-to-cell communication system that coordinate virulence and gene regulation through specific signal molecules that enable bacteria to adapt to changing conditions. Targeting this QS system reduces microbial virulence without disabling growth, thereby counteracting microbial resistance (Maeda et al., 2012; Zhong and He, 2021) brought about by selective pressure through overuse and mishandling of antipathogenic drugs (Borges and Simoes, 2019; Lewis, 2020; Zhong and He, 2021). Current therapies are limited and this situation has paved for the discovery of new antipathogenic agents (Duo et al., 2010). Recent studies have shown that plant metabolites offer essential agents to target drug-resistant microorganisms (Lee et al., 2018).

Among the promising sources of QSI (Quorum Sensing Inhibition) agents is a group of unexplored plants, the ethnobotanicals. These are plants that are utilized by ethnic groups for the treatment of diseases. Majority of the ethnic communities are geographically isolated and mostly depend on natural products for their medicines. One of the diverse ethnic communities in the Philippines is the *Ilongot-Eǵongot*. As one of the significant ethnic groups in the Philippines, they largely reside in the biologically diverse areas of Aurora, in the island of Luzon, Philippines. These areas comprise a deep, vast source of plants for medicinal use. Their use of ethnobotanicals, typical of the other ethnic tribes, are transferred from one generation to another, and thus, of cultural importance. Until recently, ethnobotanical evaluations are limited and this represents a recent group of plants that have gained interest for evaluating antipathogenic sources. The prospects of discovering natural QSI compounds from ethnobotanicals are evident in few existing researches that provide scientific validation of its potential use. These plants are powerful sources of natural QS inhibitors essential for the development of safe, novel therapeutic and antipathogenic agents (Fernando and Judan Cruz, 2020). Recently, the *Ilongot-Eǵongot* ethnobotanicals evaluated in this study have been demonstrated to possess QS inhibition properties against pathogenic bacteria such as *Pseudomonas aeruginosa* (Velasco et al., 2020; Santos et al., 2021), *Staphylococcus aureus* (Salamanca et al., 2019), *Aeromonas hydrophila* (Fernando and Judan Cruz, 2020), and *Streptococcus agalactiae* (Fernando et al., 2020). QSI actions of these plants against pathogenic fungi such as *Candida* have not yet been explored.

The formation of biofilm in pathogens is mediated by a network of genetic mechanisms. Among the key genes that are linked to biofilm adhesion, dispersion and regulation in *C. albicans* are the *Bcr1* and *Hsp90*. Expression of these genes impacts the formation and quality of the biofilm. *Bcr1* is among a system of transcription regulators that facilitates the formation of biofilm in *C. albicans* (Nobile et al., 2006; Mayer et al., 2013). As a transcription regulator, *Bcr1* directs functionally associated target genes that can eradicate a function that is carried out by redundant genes (Fanning et al., 2012). *Bcr1* and its downstream genes are expressed during adhesion of *C. albicans* on the substrate (Nobile et al., 2006) and this adhesion impacts the arrangement of the polysaccharide matrix (Douglas, 2003). The heat shock proteins (HSPs) unique to fungi and not present in humans have surfaced as a promising drug target for *C. albicans* management (Mayer et al., 2013). *HSP90*, a major HSP, is a key regulator in biofilm formation and virulence by suppressing dispersion (Robbins et al., 2011) and intricate cell signals (Pearl and Prodromou, 2006; Shapiro et al., 2009). *HSP90* also controls temperature- dependent morphogenesis by suppressing *cAMP-PKA* signals (Robbins et al., 2011). It also allows for the emergence of resistance to majority of existing antifungals (Robbins et al., 2011). Downregulation of these genes affects the formation, adherence and dispersion of the complex biofilm and its multi- dimensional polysaccharide matrix (Douglas, 2003). Hence, by negatively affecting *Bcr1* and *Hsp90* expression, fungal communication will be inactivated and consequently, virulence (Rasmussen and Givskov, 2006).

For a more efficient delivery of anti-pathogenic drugs from the natural metabolites, nanotechnology has gained substantial interest and relevance in drug design. Nanoparticles are used in drug delivery for an efficient transport of soluble drugs (Kamat et al., 2002; Daniel and Astruc, 2004) targeted to a specific site and bioavailability. The use of biosynthesized nanoparticles to enhance treatment of diseases increases the relay of drugs and subsequently enhances treatment of diseases due to their reduced dimensions, its efficiency due to their extremely small size and large relative surface area (Hentzer et al., 2003; Srisawat, 2007; Khatami et al., 2017).

This study evaluated the QSI properties of the ethnobotanical crude extracts as well as the biosynthesized nanoparticles using the *Ilongot-Eǵongot* ethnobotanicals to control biofilm formation and QS-related gene expression.

## Materials and Methods

### Collection of Plant Samples and Ethanol Extraction Procedure

Ethnobotanicals surveyed by Balberona et al. (2018) at the *Ilongot-Eǵongot* community of Maria Aurora, Aurora, Philippines were evaluated. Necessary permits from the provincial and tribal chieftains as well as from the Department of Natural Resources (DENR), Philippines were obtained for the collection of plant samples. Voucher specimens were identified by an expert taxonomist and deposited at the Department of Biological Sciences, Science City of Muñoz, Nueva Ecija, Philippines. Plant samples were collected, sterilized, air-dried and ground. Fifty grams (50 g) of ground leaf were soaked in 500 ml of 80% ethanol in a covered flask for 72 h and was filtered. The alcohol was removed through a rotary evaporator. The crude extracts were sterilized by centrifugation of the mixture at 10,000 × g for 30 min followed by membrane filtration using Acrodisc 25 mm Syringe Filter. The sterile extracts were kept at 2–8°C prior to use (Srisawat, 2007). Plant extracts evaluated were: *Hydrocotyle vulgaris* (leaf), *Eluesine indica* (root), *Eluesine indica* (leaf), *Mikania micrantha* (leaf), *Dillenia philippinensis* (leaf), *Dillenia philippinensis* (bark), *Ceiba pentandra* (leaf), *Cymbopogon winterianus* (leaf), Senna alata (leaf), *Urena lobata* (leaf), *Premna odorata* (bark), *Premna odorata* (leaf), *Stachytarpeta jamaicensis* (leaf), *Diplazium esculentum* (leaf), and *Phyllanthus urinaria* (leaf).

### Biological Synthesis of Gold Nanoparticles

Gold chloride (Sigma Aldrich) was prepared at the 10^–3^ M concentration with sterilized Milli-Q (Merck) water. For the synthesis, 5 ml of crude extract was mixed with 45 ml 10^–3^ M gold chloride. The bottles were incubated in dark condition under room temperature with consistent stirring through a magnetic stirrer for 60 min until a pink red color was attained. This change in color indicates the synthesis of AuNPs. To assess the stability of nanoparticles, the AuNPs obtained from the solution were purified by centrifugation at 4,000 rpm for 20 min and dispersed in deionized water. The water suspended NPs were frozen at 30°C overnight and were kept under vacuum for 24 h for drying.

### Preparation of Fungal Culture

Pure culture of *C. albicans* ATCC 9002 was obtained from UPLB BIOTECH, Los Baños, Laguna, Philippines. Corn meal agar was used to revive and maintain cultures of *C. albicans*. For the subsequent assays, 24 h fungal culture was suspended in sterile saline solution (0.9% NaCl) at 26–30°C and the turbidity was adjusted to 1.0 M McFarland standard.

### Analysis of Antifungal Activity of Crude Extracts (CEs) and Biologically Synthesized Gold Nanoparticles (CEs + AuNPs) Against *Candida albicans*

As a pre-screening for the biofilm formation assay, antifungal activity was assessed. The absence of zone of inhibition is required for the subsequent biofilm formation assay to rule out antifungal-mediated decrease in virulence factor production (Fernando and Judan Cruz, 2020; Velasco et al., 2020). Samples with antifungal activities shall not be included in the biofilm formation assay. The protocol of Fernando and Judan Cruz (2020) was used with some modifications. Sterile paper discs (5 mm) were soaked and air dried on sterilized petri plates under a biosafety laminar flow. Prepared media of corn meal agar on petriplates were swabbed with fungal culture. Air dried discs with treatments were seeded on plates; Ketoconazole served as positive control whereas sterile distilled water served as negative control. Plates were incubated at 37°C for 24–48 h and were observed for the appearance of the zone of inhibition.

### Microtiter Plate Biofilm Formation Assay

The effect of CEs and CEs + AuNPs on biofilm formation was measured using a microtiter plate assay. Overnight cultures of *C. albicans* with a volume of 180 μl were added with 20 μl of each treatment and were transferred to 96-well microtiter plates and incubated at 37°C for 40 h without shaking. After the incubation period, the microtiter plates were washed with sterile distilled water five times to discard planktonic cells. These were air dried for 45 min and stained with 150 μl of 1% crystal violet for 45 min (Fernando and Judan Cruz, 2020). Plates were rinsed with sterile distilled water five times.

To quantify the biofilm, 200 μl of 95% ethanol was added to destain the wells. From each well, 100 μl were transferred to a new microtiter plate. Optical Density (OD) values were determined at 595 nm (Djordjevic et al., 2002) using UV-visible spectrophotometer (Biotek Instruments, Inc., United States).

### Gene Expression Analysis

Treatments with significant inhibition in biofilm formation were subjected to gene expression analysis. RNA extraction was done following the RNA extraction kit manufacturer’s protocol (Promega Corp.) with modifications. The expression of *HSP90* and *Bcr1* in *C. albicans* was determined through qRT-PCR analysis using Bio-Rad CFX96 Real-Time System Thermal Cycler and KAPA One Step RT-PCR kit (KAPA Biosystems). The specific primers used were: *HSP90*F 5′ CGATGAATATGCCATGACT, *HSP90*R 5′ TCCATAGCAGATTCTCCAG 3′ (Mi-Kyung et al., 2004); *Bcr1*F 5′ GGCTGTCCATGTTGTTGTTG 3′, *Bcr1*R 5′ GAGCACGCATCTATGGCTTA 3′ (Alves et al., 2000); and the internal standard *16S*F 5′ATGGCCGTTCTTAGTTGGTG 3′, *16S*R 5′ GCCAAGGCTTATACTCGCTG 3′ (Zhang et al., 2000). The qRT-PCR program was as follows: incubation at 42°C for 5 min for reverse transcription; 95°C for 1 min; followed by 45 cycles of 95°C for 10 s, 60°C for 30 s, and 72°C for 20 s (Nailis et al., 2006).

### Statistical Analysis

Significant differences in OD values were analyzed via independent sample Tukey’s Honest Significant Difference Test with 0.05 level of significance. For the gene expression analysis, Ct values were analyzed using the 2^–ΔΔ*CT*^ (Livak) method (Livak and Schmittgen, 2001). The statistical analysis for the gene expression was performed with the use of Kruskal-Wallis test (non-parametric ANOVA).

## Results

### Antifungal Activity of CEs and CEs + AuNPs Against *C. albicans*

None of the CEs and CEs + AuNPs showed antifungal activity against *C. albicans*, hence, all samples were evaluated for its effects on biofilm formation.

### Inhibitory Effect of CEs and AuNPs on Biofilm Formation of *C. albicans*

The optical density (OD) values of the *C. albicans* clinical isolate culture treated with 13 CEs namely *H. vulgaris* leaf (0.065 mg/ml); *M. micrantha* leaf (0.062 mg/ml); *C. pentandra* leaf (0.066 mg/ml); *C. winterianus* Leaf (0.063 mg/ml); *S. alata* (0.063 mg/ml); *U. lobata* leaf (0.065 mg/ml); *D. philippinensis* leaf (0.080 mg/ml); *P. odorata* bark (0.060 mg/ml); *S. jamaicensis* leaf (0.067 mg/ml)*; E. indica* roots (0.067 mg/ml); *D. esculentum* (0.083 mg/ml)*; E. indica* L. leaf (0.067 mg/ml); and *P. urinaria* L. (0.067 mg/ml) showed significantly lower OD values in biofilm formation compared to the negative control (no extract) with a value of 0.19 mg/ml (Table 1). In contrast, two (2) CEs showed significantly higher OD values when compared to the control: *D. philippinensis* (0.12 mg/ml) bark and *P. odorata* leaf (0.19 mg/ml). These showed no QSI activity with increased formation of biofilm.

**TABLE 1:** Biofilm inhibition in *C. albicans* as affected by CEs and CEs + AuNPs.

Scientific name	Crude extract	Biologically synthesized gold nanoparticles
*H. vulgaris*	0.065*^a^	0.080*^b^
*M. micrantha*	0.062*^a^	0.067*^b^
*D. philippinensis* (bark)	0.116	0.066*
*C. pentandra*	0.066*	0.066*
*C. winterianus*	0.064*	0.066*
*S. alata*	0.063*	0.062*
*U. lobata*	0.065*	0.065*
*D. philippinensis* (leaves)	0.080*	0.135
*P. odorata* (bark)	0.06*^a^	0.082*^b^
*S. jamaicensis*	0.067*	0.074*
*E. indica* (roots)	0.066*	0.068*
*P. odorata* (leaves)	0.186	0.072*
*D. esculentum*	0.083*^a^	0.072*^b^
*E. indica* (leaves)	0.067*	0.070*
*P. urinaria*	0.067*	0.071*
Control	0.190	0.190

In columns, (*) indicates significantly lower O.D. value in biofilm formation compared the control; Different letter superscripts among rows indicate significant difference.

The OD values of 14 CEs + AuNPs showed significant decrease in *C. albicans* biofilm formation compared to the control (Table 1) with the following values: *H. vulgaris* (0.080 mg/ml); *M. micrantha* leaf (0.067 mg/ml); *D. philippinensis* bark (0.067 mg/ml); *C. pentandra* leaf (0.067 mg/ml); *C. winterianus* leaf (0.067 mg/ml); *S. alata* (0.063 mg/ml); *U. lobata* leaf (0.065 mg/ml); *P. odorata* bark (0.082 mg/ml); *S. jamaicensis* leaf (0.074 mg/ml); *E. indica* (0.067 mg/ml); *P. odorata* leaf (0.072 mg/ml); *D. esculentum* (0.073 mg/ml); *E. indica* leaf (0.067 mg/ml); *P. urinaria* leaf (0.071 mg/ml).

### Downregulation of *Bcr1* and *HSP90* as Affected by CEs and CEs + AuNPs

**FIGURE 1:**
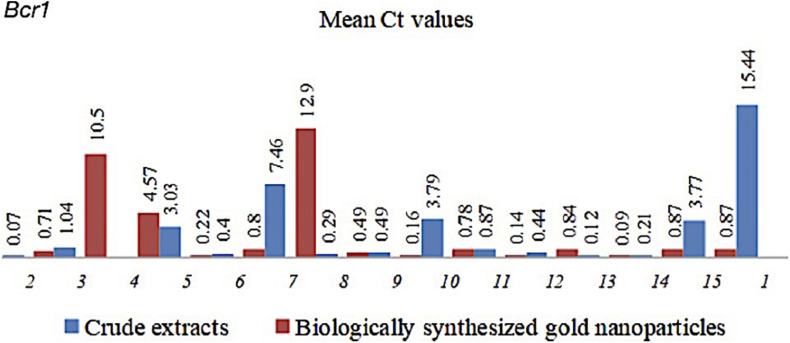
Mean Ct values of *Bcr1* in *C. albicans* with CEs and CEs + AuNPs. (1) Control (2) *H. vulgaris* (3) *M. micrantha* (4) *D. philippinensis* (bark) (5) *C. pentandra* (6) *C. winterianus* (7) *S. alata* (8) *U. lobata* (9) *D. philippinensis* (leaf) (10) *P. odorata* (bark) (11) *S. jamaicensis* (leaf) (12) *E. Indica* (root) (13) *E. esculenta* (14) *E. indica* (leaf) (15) *P. urinaria*.

The CEs and CEs + AuNPs that showed significantly lower biofilm formation were subjected to gene expression analysis. *Bcr1* expression analysis showed down regulation in all CEs and CEs + AuNPs treatments with significantly lower biofilm formation in the virulence assay (Figure 1). The expression of *Bcr1* in *C. albicans* was significantly downregulated in association with the CEs of *H. vulgaris* (0.071), *M. micrantha* leaf (1.036), *C. pentandra* leaf (3.033), *C. winterianus* Leaf (0.403), *S. alata* (7.459), *U. lobata* leaf (0.292), *D. philippinensis* leaf (0.485), *P. odorata* bark (3.792), *S. jamaicensis* leaf (0.87), *E. indica* roots (0.437), *D. esculentum* (0.115), *E. indica* leaf (0.213), and *P. urinaria* leaf (3.772) as compared to the control with no plant extract used (15.44). Significant downregulation of the *Bcr1* gene was also observed in CEs + AuNPs of *H. vulgaris* (0.711), *M. micrantha* (10.496), *D. philippinensis* bark (4.567), *C. pentandra* leaf (0.223), *C. winterianus* Leaf (12.898), *S. alata* (0.799), *U. lobata* leaf (0.490), *P. odorata* bark (0.161), *S. jamaicensis* leaf (0.780), *E. indica* roots (0.140), *P. odorata* leaf (0.835), *D. esculentum* (0.086), *E. indica* leaf (0.87), and *P. urinaria* (0.87) (Figure 1).

**FIGURE 2:**
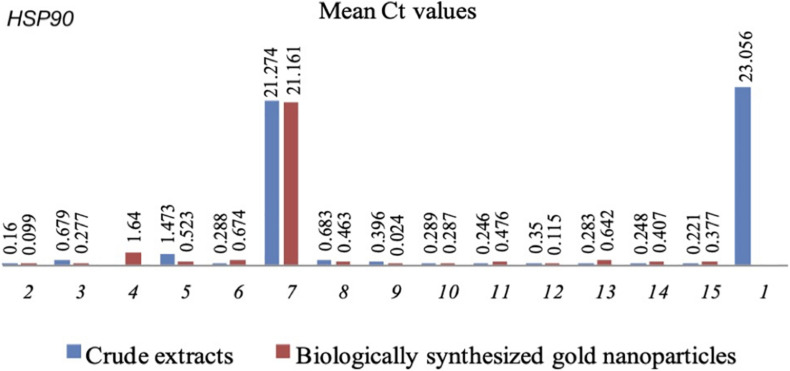
Mean Ct values of *HSP90* in *C. albicans* with CEs and CEs + AuNPs. (1) Control (2) *H. vulgaris* (3) *M. micrantha* (4) *D. philippinensis* (bark) (5) *C. pentandra* (6) *C. winterianus* (7) *S. alata* (8) *U. lobata* (9) *D. philippinensis* (leaf) (10) *P. odorata* (bark) (11) *S. jamaicensis* (leaf) (12) *E. Indica* (root) (13) *E. esculenta* (14) *E. indica* (leaf) (15) *P. urinaria*.

*HSP90* also showed down regulation in *C.albicans* treated with CEs and CEs + AuNPs that showed lower biofilm formation values. The expression of *HSP90* was significantly downregulated in association with the CEs of *H. vulgaris* (0.16), *M. micrantha* leaf (0.679), *C. pentandra* leaf (1.473), *C. winterianus* leaf (0.288), *S. alata* (21.274), *U. lobata* leaf (0.683), *D. philippinensis* leaf (0.396), *P. odorata* bark (0.289), *S. jamaicensis* leaf (0.246), *E. indica* roots (0.350), *D. esculentum* (0.283), *E. indica* leaf (0.248), and *P. urinaria* (0.221) as compared to the control with no plant extract used (23.056) (Figure 2). Significant downregulation of the *HSP90* was also observed in CEs + AuNPs of *H. vulgaris* (0.099), *M. micrantha* (0.277), *D. philippinensis* bark (1.640), *C. pentandra* leaf (0.523), *C. winterianus* Leaf (0.674), *S. alata* (21.161), *U. lobata* leaf (0.463), *P. odorata* bark (0.024), *S. jamaicensis* leaf (0.287), *E. indica* roots (0.476), *P. odorata* leaf (0.115), *D. esculentum* (0.462), *E. indica* leaf (0.407), and *P. urinaria* (0.377) (Figure 2).

## Discussion

Inhibition of biofilm formation by the ethnobotanical CEs and CEs + AuNPs in this study may be attributed to the presence of known QSI agents that are recognized to negatively affect signal receptors (Kalia, 2013) responsible for the functional communication between adjacent cells (Miller and Bassler, 2001). The *Ilongot-Eǵongot* ethnobotanicals evaluated in this study are known to possess active groups of metabolites against QS such as: flavonoids, saponins and tannins in *C. pentandra* (Friday et al., 2011); flavonoid, saponins, tannins, alkaloids, and geranoil in *C. winterianus* (Anosike et al., 2012); *S. alata* contains flavonoid, saponins, tannins, alkaloids, and terpenes (Sule et al., 2011), *U. lobata* with saponins, tannins, alkaloid, and terpenoid (Fagbohun et al., 2012), and *P. odorata* with flavonoid, saponins, and terpenoids (Chichioco-Hernandez and Paguigan, 2009). The isolated terpene and sterol compounds in *C. pentandra* attenuated virulence factors in *P. aeruginosa* (Muñoz-Cázares et al., 2017). Only the major metabolites have been evaluated and reported against QS while the specific bioactive components of the ethnobotanicals in this study have not yet been elucidated and presents an avenue for research in detailed phytochemistry.

The well documented mechanism of QSI action of phytochemicals is linked to their similarity in the chemical structure to QS signals and to their capacity to suppress signal receptors (Kalia, 2013). Plants have been known to contain phytochemicals associated with QSI activities and is considered as one of the most powerful natural sources of isolated QSI compounds. These compounds can reduce microbe pathogenicity (Rasmussen and Givskov, 2006) owing to their ability to block in intra and inter-species QS communication systems (Teplitski et al., 2000). This ability of natural compounds to suspend QS systems can serve as a defense strategy to fight against microbial penetration. As a prospective source of antivirulence agents (Rawat et al., 2016) that are safe for human health, it owes its advantage to its chemical stability and highly effective low-molecular-mass molecules (Rasmussen and Givskov, 2006) with non-toxic inhibitors of QS (Hentzer et al., 2003).

Evaluating the effects of potential QSI agents on the molecular mechanisms directing biofilm formation is a critical strategy to facilitate advances in novel antivirulence therapies. In this study, the expression of 2 biofilm-linked genes, *Bcr1* and *HSP90*, as affected by CEs and CEs + AuNPs were evaluated. Molecular expression analyses showed downregulation of both *Bcr1* and *HSP90* as affected by CEs and CEs + AuNPs. Expression of *Bcr1* and its downstream genes influences adhesion and arrangement of the polysaccharide matrix in *C. albicans*. Hence, downregulation of *Bcr1* affects the formation of the complex biofilm and its multi- dimensional polysaccharide matrix (Douglas, 2003); this means that biofilm formation will be repressed or will not yield a thick extracellular matrix. On the other hand, by targeting *HSP90* downregulation, dispersion will be suppressed and cell signals critical to biofilm formation will be blocked without developing resistance to existing antifungals (Robbins et al., 2011). The compounds in CEs and CEs + AuNPs may have acted as QSI molecules that have blocked the pathway of *Bcr1* and *HSP90*, hence, its downregulation. This showed that the production of QS molecules was reduced and have decreased in the expression of a specific receptor or transcription factor (Nobile et al., 2006). It has been recognized that expression of QS genes is important in the production of virulence factors such as the formation of biofilm, and this information gives improved understanding of the function of the genes associated with its morphological features (Nobile et al., 2006). Thus, downregulation of *Bcr1* and *HSP90* by the CEs and CEs + AuNPs not only have the potential to inhibit the growth of biofilm but also that of antifungal resistance. Blocking or minimizing expression of these genes provides a key strategy to developing drugs for *C. albicans* management.

The efficiency on the use of nanoparticles was demonstrated in this study wherein treatments with CEs + AuNPS showed significantly lower biofilm formation in comparison with CEs alone. The CEs + AuNPs conjugation length and intensities decreased from 595 to 544 nm which signifies the decrease in size. The study of Emmanuel et al. (2017) demonstrates that the decrease in the conjugation length and intensities of AuNPs indicates the decrease in particle size. The formation of CEs + AuNPs in this study were monitored by analyzing the excitation due to the applied electromagnetic field of Surface Plasmon resonance (SPR) and absorption values. SPR peaks attained in UV–vis spectroscopy is one of the versatile techniques to confirm the formation of metal NPs and was generated due to the coherence of electrons on the surface of AuNPs. The shift to the blue or red in the λmax of the SPR peak could be related to the obtained morphology of NPs that has various shapes, sizes or extract dependencies of formed AuNPs (Vellaichamy and Periakaruppan, 2016; Emmanuel et al., 2017; Kanwal et al., 2018). The color change in reaction from yellowish to pink red and decreased conjugation length confirms the formation of CE + AuNPs (Mukherjee et al., 2016; Ovais et al., 2016). Furthermore, the pH of the solution increased from 6.0 to 6.5 after the addition of the crude ethnobotanical extracts indicating to a more stable state of the gold nanoparticles. The stability of gold nanoparticles is pH-responsive (Park et al., 2019) and its stability is pH-dependent, as shown in studies using natural compound for its synthesis (Tyagi et al., 2011).

The development of methods for integrated solution and control of pathogenic virulence and drug resistance has led many scientists to evaluate nanotechnology for efficient delivery of anti-pathogenic drugs from the natural compounds. Since its revolution, nanotechnology has been used to improve the uptake of soluble drugs (Ould-Ouali et al., 2005) due to their extremely reduced dimensions and somewhat large surface area (Kamat et al., 2002). Its safety also accompanies its advantages as it produces environmentally non-toxic molecules (Khatami et al., 2017). The results of this study may indicate expedited delivery system of the compounds through extremely reduced particle size and increased surface area that facilitates entry of compounds to the phospholipid- and glycoprotein-embedded cell membrane (Cabrera et al., 2017).

This study has shown that ethnobotanicals are a promising source of antipathogenic agents. Except for a few studies, these plants largely remain unexplored for their pharmacological potential. A number of studies have shown proof that ethnobotanicals possesses QSI actions in virulence factors in bacteria such as biofilm formation (Judan Cruz et al., 2018; Fernando and Judan Cruz, 2020; Fernando et al., 2020; Velasco et al., 2020; Santos et al., 2021), coagulase (Vias et al., 2018; Salamanca et al., 2019), pyocyanin production (Barrogo et al., 2018; Limos et al., 2018a), swarming motility (Barrogo et al., 2018; Limos et al., 2018a; Padilla et al., 2018), DNase (Limos et al., 2018b), and α- Hemolysin (Limos et al., 2018b; Vergara et al., 2018), showing the immense prospects of tapping these plants for antivirulence drug design. The QSI actions of the ethnobotanicals were confirmed through expression analyses of QS-linked genes such as *lasR, rhlR, ahyR*, and *agrA.*

Targeting virulence factors is a promising approach to design new and effective antifungal therapies (Mayer et al., 2013). Biofilm is one of the QS-regulated virulence factors that contribute to the pathogenicity as well as to the increasing development of fungal drug resistance in *C. albicans*. Despite the existing antifungal drugs, fungal resistance is evolving due to long term exposure. A novel approach for its control is to obtain plant bioactive compounds to create a wide variety of drugs (Cruz et al., 2007) that targets the formation of biofilm. Recently, a number of antifungal drugs have been designed to contain natural derivatives or compounds mimicking natural products (Newman, 2008). In *C. albicans*, numerous plant extracts and its compounds are already known to change its adhesion mechanics (Ahmad and Aqil, 2007); adhesion being the first step in its biofilm formation and significantly contributes to *C. albicans* pathogenicity (Mukherjee et al., 2003).

Diverse natural products are recognized to inhibit biofilm formation through scientific validations and studies. Therefore, the discovery of plant bioactive compounds that controls pathogenicity becomes a fundamental strategy (Ahmad and Aqil, 2007) toward *C. albicans* management. This paper highlights the great pharmacological potential of these ethnobotanical extracts for developing efficient therapeutic agents against *C. albicans* without the risk of developing drug resistance. This potential can be further improved through nanotechnology. This new understanding can be used to direct the discovery of novel approaches for preventing and controlling complex and resistant biofilms.

## Data Availability Statement

The original contributions presented in the study are included in the article/supplementary material, further inquiries can be directed to the corresponding author/s.

## Author Contributions

KJC provided the concept and design of the study, wrote the first and final drafts of the manuscript, and performed laboratory works. EA wrote the first draft of the manuscript, performed laboratory works, and provided laboratory materials. SF performed laboratory works, wrote a section of the manuscript, and performed statistical analyses. KW wrote a section of the manuscript, provided a portion of the laboratory funding, and supervised laboratory works. All authors contributed to manuscript revision, read, and approved the submitted version.

## Conflict of Interest

The authors declare that the research was conducted in the absence of any commercial or financial relationships that could be construed as a potential conflict of interest.

## Dedication

This work is dedicated to the *Ilongot-Eǵongot* ethnic community of Maria Aurora, Aurora, Philippines.
